# Supplementary material for: Ocean cavity regime shift reversed West Antarctic grounding line retreat in the late Holocene
Source: Nat Commun. 2024 Apr 23;15:3176. doi: 10.1038/s41467-024-47369-3 (PMC11039676; doi:10.1038/s41467-024-47369-3)
Supplement: Supplementary file 1 — Supplementary Information [file 41467_2024_47369_MOESM1_ESM.pdf]

Supplementary information for:

Lowry, D.P., Han, H.K., Golledge, N.R., Gomez, N., Johnson, K.M., & McKay, R.M. (2024) Ocean cavity regime shift reversed West Antarctic grounding line retreat in the late Holocene, *Nature Communications*.

Ice sheet modelling with simple viscoelastic Earth deformation

The environmental forcings we use to force the model in the deglacial experiments are shown in Fig S1 (20 to 0 ka BP). The global mean sea level forcing is based on a compilation of global sea level proxy records from the last deglaciation (*1*-*4*). Surface temperature (°C) and precipitation (%) anomalies are derived from the West Antarctic Ice Sheet (WAIS) Divide ice core reconstruction (*5*), and applied as anomalies to the modern climatology (*6*). The ocean temperature (°C) anomalies are derived from the TraCE-21ka deglacial climate model simulation (*7*, *8*), and are applied to a spatial field of ocean temperatures optimised for modern basal ice shelf melt rates (*9*).

Our model benchmarking focuses on the deglacial ice sheet evolution (Fig S2). Parameter values for our reference simulations are shown in Table S1. Last Glacial Maximum ice volume of our reference simulations ranges from 10.9 to 11.5 m sea level equivalent (s.l.e.) above the present-day ice sheet (Fig S2a), within the range of estimates of various studies, i.e. 7.3 to 13.6 m s.l.e. (*10*-*12*). Deglacial ice thickness changes are within the ranges indicated by cosmogenic geochronology in the Transantarctic Mountains (*13*-*16*), with rapid deglacial ice thinning occurring in the models from the early to middle Holocene (Fig S2b,c,d).

We also run a present-day reference simulation, in which we map ice temperature fields, internal velocities and bed conditions from our model spin-up to the present-day ice sheet configuration (*17*), and run forward in time using historical climate forcing from NorESM1-M (*18*). This produces reasonable fit to observations of grounding line position, ice thickness (Fig S3a,b) and ice surface velocity (Fig S3c,d). Bias with respect to ice surface velocity does occur at the Siple Coast of Antarctica, with lower-than-observed surface velocity of West Antarctic ice streams and regions of higher-than-observed ice shelf velocity. Modern basal melt rates are within the ranges estimated by Adusumilli et al. (2020) (*19*), with modelled melt rates as high as 18 m yr$^{-1}$ in the Amundsen Sea sector ice shelves, but generally < 1 m yr$^{-1}$ for the Ross Ice shelf, which overlies a cold ocean cavity (Fig S3e). We extend this run for 500 years to demonstrate the ice sheet response to present-day climate over a longer duration. Sea level equivalent ice volume shows a small decrease (Fig S3f), as

expected given modern Antarctic mass loss, but grounding line position remains relatively unchanged at the Siple Coast (gold line in Fig S3b,d).

Using the standard ocean forcing from TraCE-21ka (i.e. no mid-Holocene warming), we explore the parameter space of three solid earth parameters in the simple two-layer viscoelastic Earth deformation model from Bueler et al., (2007) (*20*): mantle viscosity, lithosphere flexural rigidity and mantle density. Experiments are run with 8 values of mantle viscosity: 1e19, 5e19, 1e20, 5e20, 7.5e20, 1e21, 2.5e21, and 5e21 Pa s; 5 values of lithosphere flexural rigidity: 1e23, 1e24, 5e24, 1e25, and 5e25 N m; and 2 values of mantle density: 3300 and 4500 kg $m^{-3}$. Mantle viscosity is an important control on the rate of isostatic rebound, with grounding-line retreat and readvance only occurring within the range of 5e20 to 1e21 Pa s (Fig S4). The relationship between grounding-line migration and lithosphere flexural rigidity is non-linear, with especially high and low values limiting grounding-line retreat. At intermediate values, in some cases (e.g. 5e24 N m in Fig S4), pinning points on the outer continental shelf limit collapse of the outer ice shelf as well as limiting grounding line retreat and readvance. Of the two mantle density values tested, the higher value delays and limits grounding line retreat and limits readvance (Fig S5).

Because large uncertainties exists with respect to the timing of WAIS retreat and advance of the Siple Coast grounding line, and because the radiocarbon input and decay modelling indicates differences in ice stream behaviour (*21*), we run additional ocean forcing experiments in which we vary the timing of onset and removal of anomalous ocean warming (Fig S7). The timing of retreat and advance of WIS and BIS corresponds to these forcings. Under anomalous ocean warming of +0.6°C from the standard TraCE-21ka forcing, the simulated ice shelf collapses between 6 and 4 ka BP in our simulations. We also explore the impact of model resolution by running an experiment at 10 km spatial resolution (as compared to our standard 20 km resolution experiments). Because increasing model resolution increases ice sheet and ice shelf velocity and produces thinner ice shelves, we ran this experiment using lower enhancement factors for the Shallow Ice and Shallow Shelf Approximations (i.e. 1.5 and 0.4, respectively). The grounding line retreats and advances in response to the anomalous ocean forcing application and removal, demonstrating that the grounding-line reversibility in response to the change in ocean forcing is robust with different model resolutions.

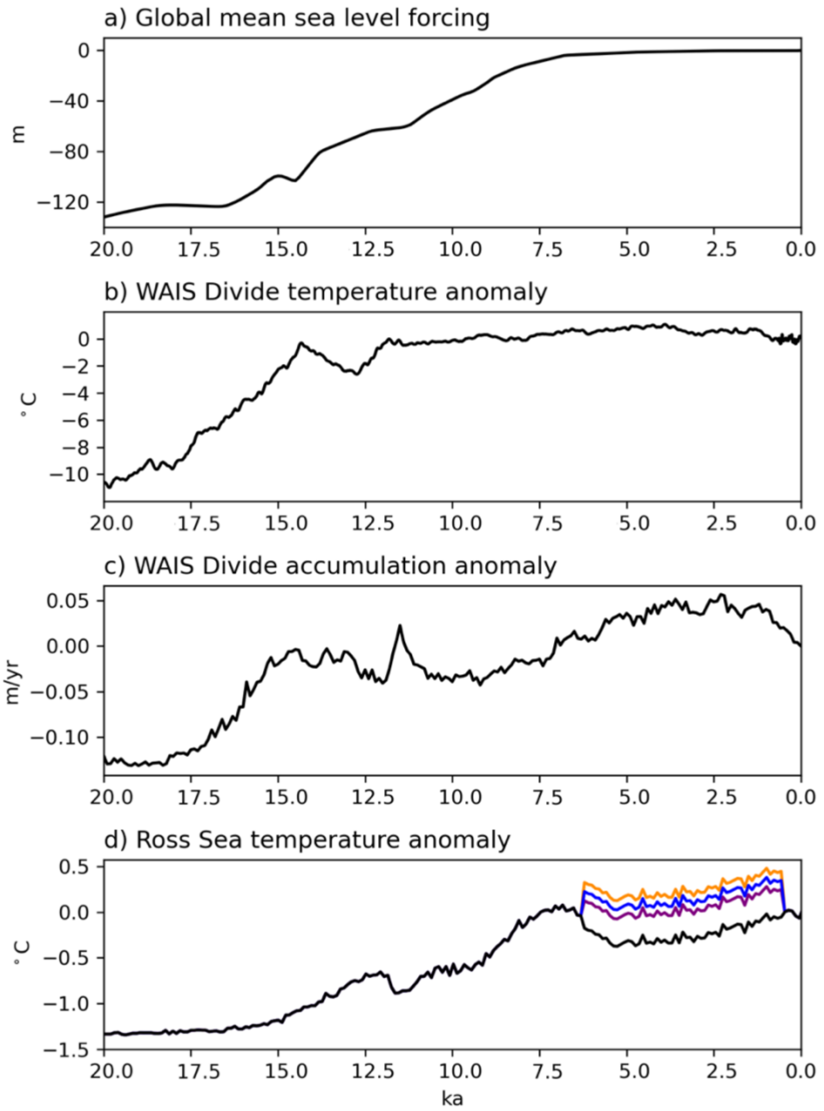


**Fig S1. Environmental forcings applied in the ice sheet model simulations.** (a) Global mean sea level forcing (m), (b) surface temperature anomaly (°C), (c) snow accumulation (m $yr^{-1}$, but applied as a % anomaly in the ice sheet model), (d) ocean temperature anomaly in the Ross Sea region. The standard TraCE-21ka ocean forcing (black line) is the anomaly at ~500m depth relative to 0 ka, and examples of modified ocean forcings (+0.3, +0.4 and +0.5°C) are shown in color (purple, blue and orange, respectively).

| Ice sheet model parameter | Parameter value | Units |
|---|---|---|
| **PISM version** | 2.0.3 | |
| **Domain x** | 289 | cells |
| **Domain y** | 249 | cells |
| **Vertical ice layers** | 121 | |
| **Vertical bedrock layers** | 20 | |
| **Computational box height** | 6000 | m |
| **Z spacing (ice)** | quadratic | |
| **Z spacing (bedrock)** | equal | |
| **Shallow-ice approximation enhancement factor** | 3.0 | |
| **Shallow-shelf Approximation enhancement factor** | 1.0 | |
| **Grounding line scheme** | Subgrid, basal melt of partially grounded cells | |
| **Pseudo plastic *q*** | 0.75 | |
| **Eigen calving *K*** | 1e17 | |
| **Thickness calving threshold** | 190 | m |
| **Till porewater overburden fraction** | 0.05 | |
| **Till friction angle minimum** | 3 | ° |
| **Till friction angle maximum** | 30 | ° |
| **Atmospheric lapse rate** | 8 | K |
| **Surface pdd positive threshold temperature** | 268 | K |
| **Surface pdd standard deviation value** | 2 | K |

**Table S1. Ice sheet model parameters.** List of Parallel Ice Sheet Model parameter values used in the reference simulations in which we vary solid Earth parameters and Holocene ocean forcing.

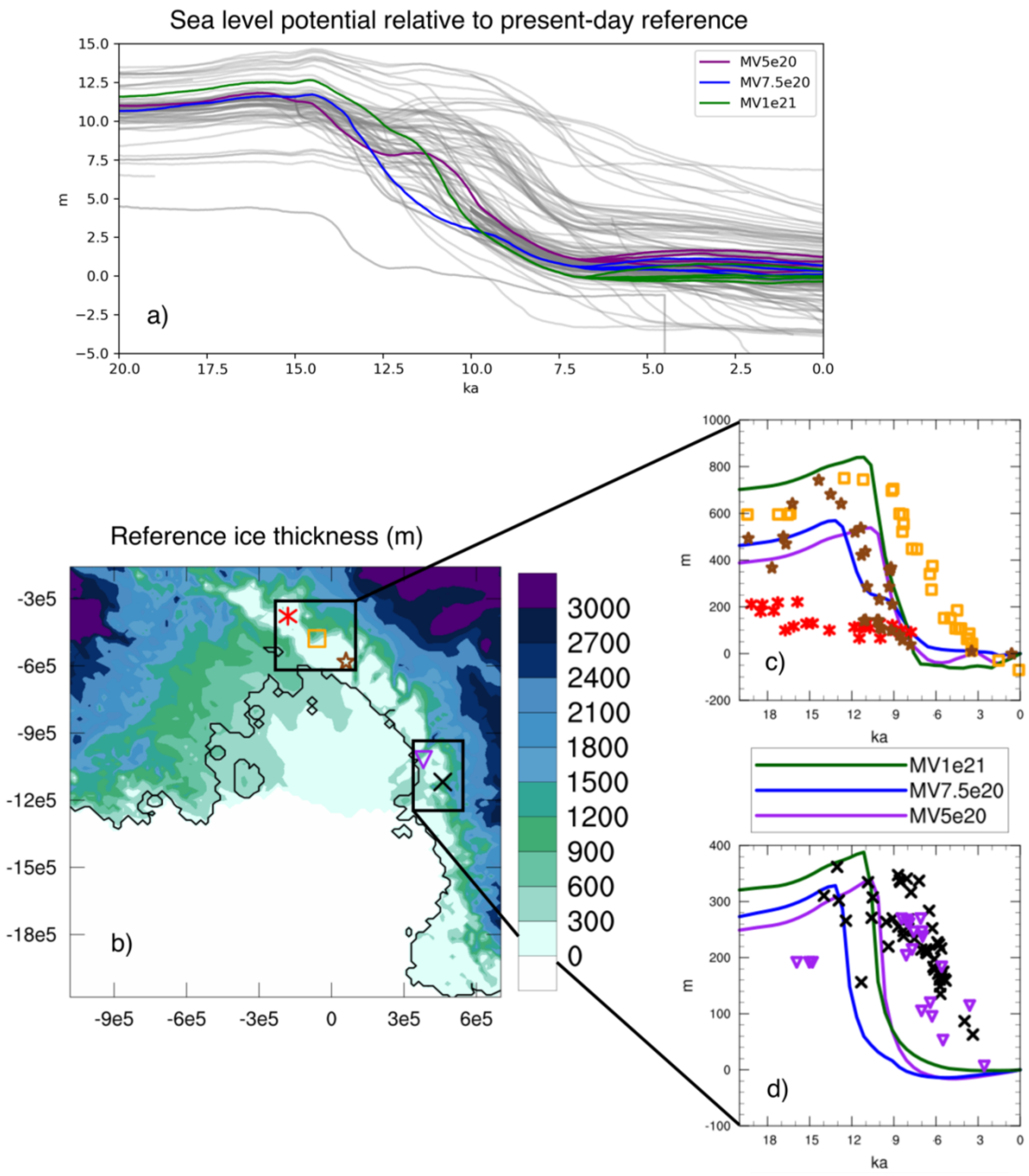


**Fig S2. Deglacial ice volume and thickness evolution.** a) Sea level potential relative to the present-day of our deglacial experiments (n=270). The impact of mantle viscosity of our reference simulations are indicated by the colored lines, and all other simulations are shown by the gray lines. b) Modern ice thickness of the present-day reference model. The colored markers indicate proxy site locations of deglacial ice thickness from Reedy Glacier (red cross; *13*), Scott Glacier (orange square; *14*), Beardmore Glacier (brown star; *14*), Byrd Glacier (purple triangle; *16*), and Darwin and Hatherton Glaciers (black x; *15*). c,d) Spatially-averaged modelled ice thickness anomalies over the last deglaciation compared to the deglacial ice thinning records.

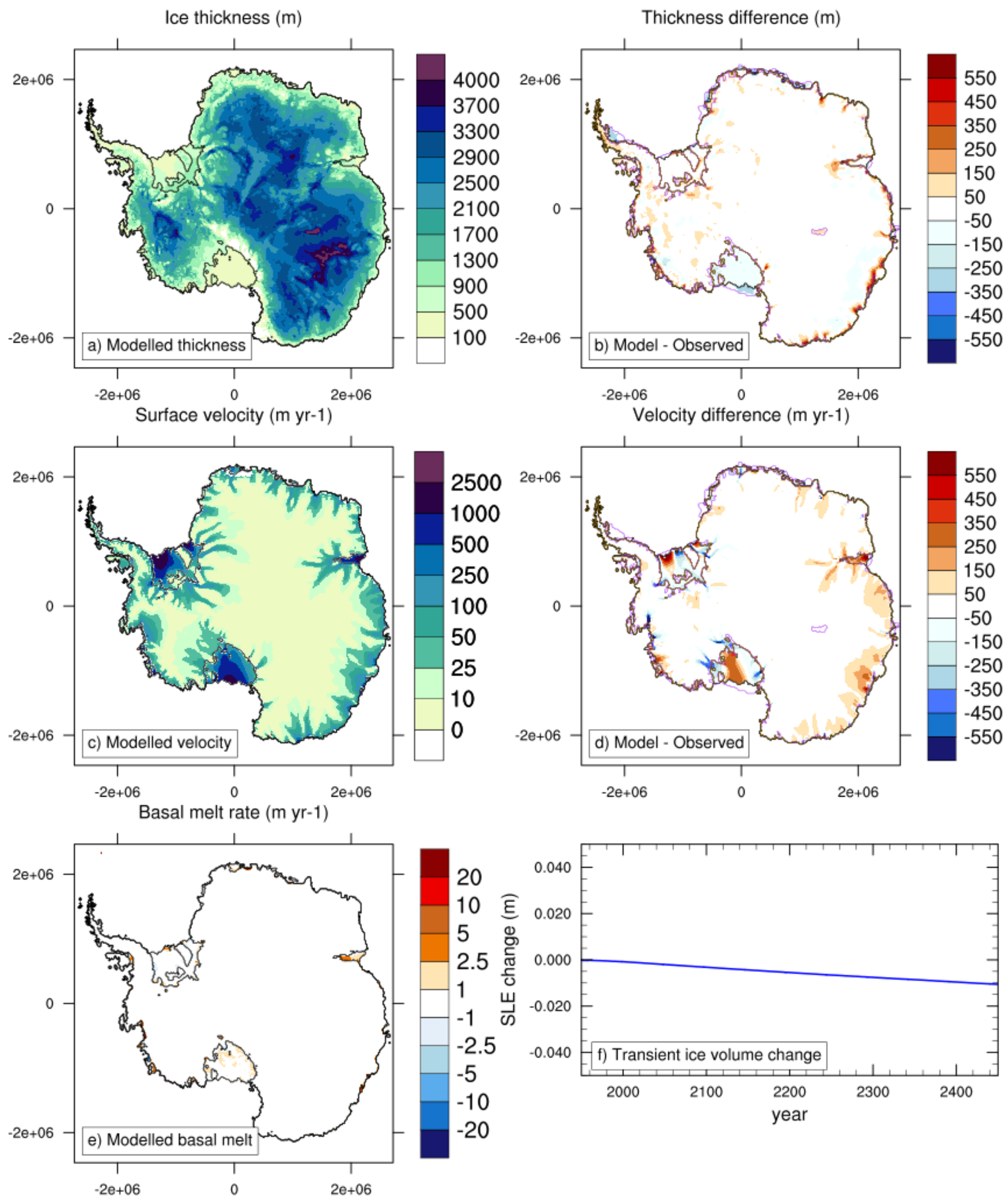


**Fig S3. Present-day ice sheet model reference simulation.** This reference simulation was run for the period 1950 to 2450 using the model parameters listed in Table S1 and climate forcing derived from NorESM1-M. Following 2015, the climate forcing is held constant using a mean present-day climate. Shown are a) modelled ice thickness at 2015 and (b) the differences to Morlighem et al. (2020) (*17*); c) modelled ice surface velocity at 2015 and d) difference to Mouginot et al. (2017) (*22*); e) Modelled basal melt at 2015. The black lines indicate the modelled ice sheet grounding and calving line positions at 2015, the dark gold lines indicate the modelled grounding line position at 2450, and the purple lines in the difference plots indicate the observed grounding and calving line positions. f) Transient ice volume change (sea level equivalent in m) relative to 1950 of the reference simulation.

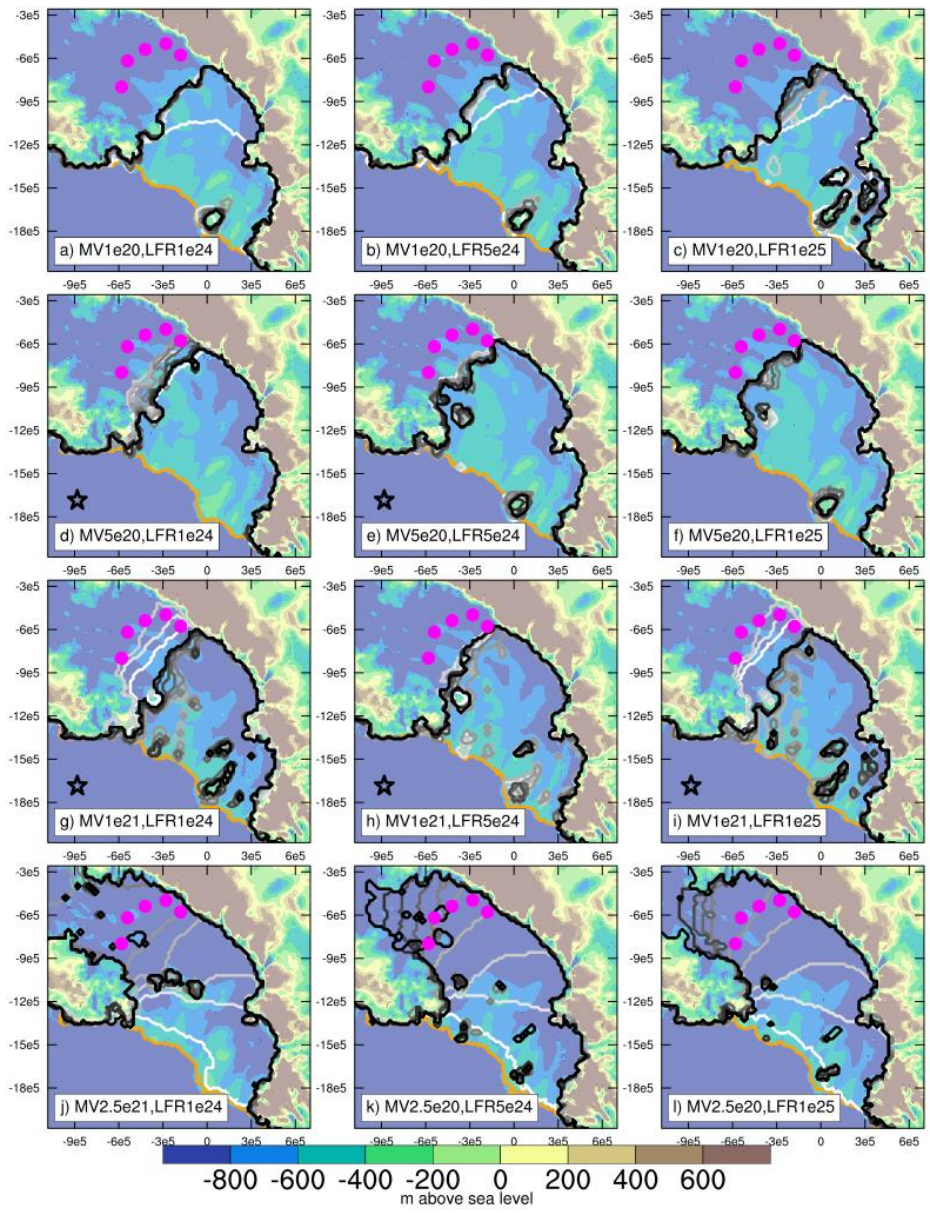


**Fig S4. Predicted Holocene grounding line migration with varying lithosphere flexural rigidity and mantle viscosity.** Bed topography (m above sea level) at 0 ka BP, and grounding line position every 1000 years from 9 ka BP (white) to 0ka BP (black), darkening in scale, for ice sheet simulations with varying mantle viscosity (MV; units of Pa s) and lithosphere flexural rigidity (LFR; units of N m): (a-c) MV of 1e20 Pa s, (d-f) MV of 5e20 Pa s, (g-i) MV of 1e21 Pa s, and (j-l) MV of 2.5e21, with LFR of 1e24, 5e24, and 1e25, respectively. The orange line indicates the glacial grounding line position at 18 ka BP. Magenta circles indicate the West Antarctic subglacial sediment sites. Stars indicate that the grounding retreats and readvances in the simulation.

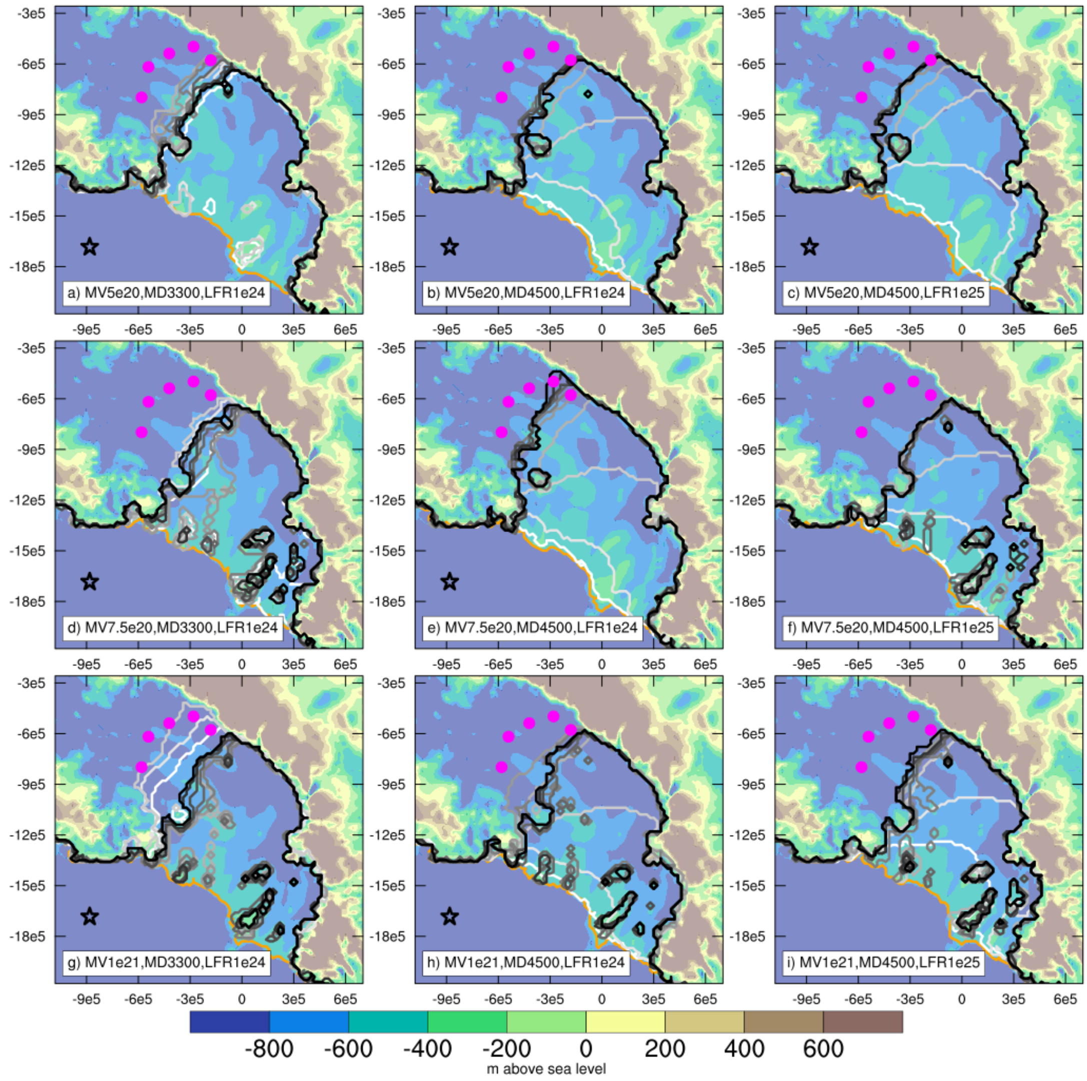


**Fig S5. Predicted Holocene grounding line migration with varying mantle density, lithosphere flexural rigidity and mantle viscosity.** Bed topography (m above sea level) at 0 ka BP, and grounding line position every 1000 years from 9 ka BP (white) to 0 ka BP (black), darkening in scale, for ice sheet simulations with varying mantle viscosity (MV; units of Pa s), mantle density (MD; units of kg $m^{-3}$) and lithosphere flexural rigidity (LFR; units of N m): (a) MV of 5e20 Pa s, MD of 3300, LFR of 1e24; (b) MV of 5e20 Pa s, MD of 4500, LFR of 1e24; (c) MV of 5e20 Pa s, MD of 4500, LFR of 1e25; (d) MV of 7.5e20 Pa s, MD of 3300, LFR of 1e24; (e) MV of 7.5e20 Pa s, MD of 4500, LFR of 1e24; (f) MV of 7.5e20 Pa s, MD of 4500, LFR of 1e25; (g) MV of 1e21 Pa s, MD of 3300, LFR of 1e24; (h) MV of 1e21 Pa s, MD of 4500, LFR of 1e24; (i) MV of 1e21 Pa s, MD of 4500, LFR of 1e25. The orange line indicates the glacial grounding line position at 18 ka BP. Magenta circles indicate the West Antarctic subglacial sediment sites. Stars indicate that the grounding retreats and readvances in the simulation.

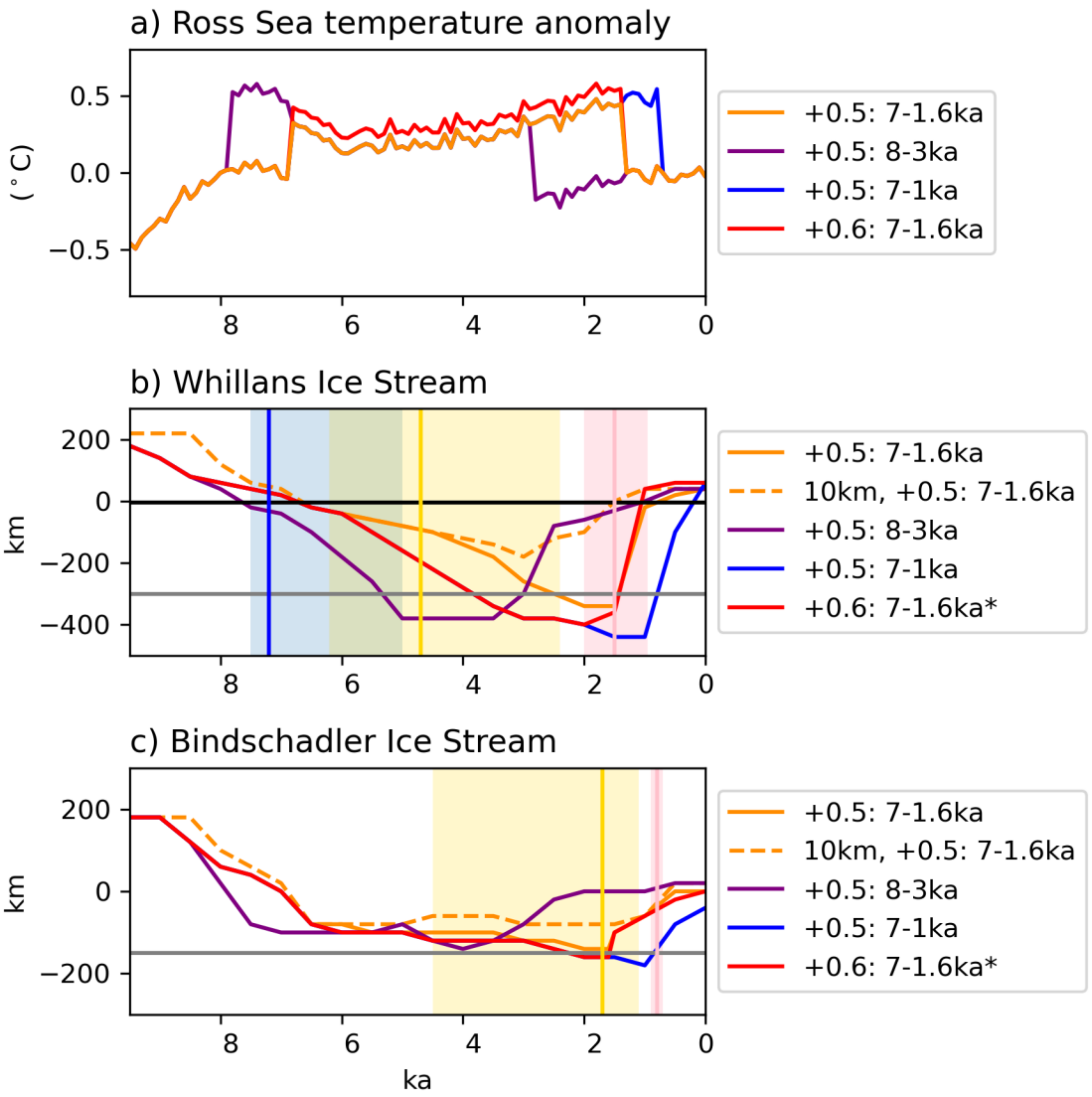


**Fig S6. Modelled grounding line position with alternative forcings and resolution.** (a) Modified TraCE-21ka ocean forcings for the Ross Sea sector, with colors corresponding to the timing and magnitude of the anomalous warming. Distance of ice sheet grounding from the modern grounding line for the (b) Whillans and (c) Bindschadler ice streams for simulations with a mantle viscosity of 7.5e20 Pa s. The dotted orange line is for a simulation using higher spatial resolution and reduced SIA and SSA enhancement factors. Horizontal lines correspond to the proxy sites. The vertical blue line shows the radiocarbon age for retreat at Whillans Grounding Zone (WGZ) from Venturelli et al. (2020), with blue shading indicating age uncertainty. The vertical yellow and pink lines respectively indicate the modelled age for retreat and advance at the WIS and BIS sites from Neuhaus et al. (2021) with colored shading indicating age uncertainty. The asterisk for the +0.6°C simulation indicates that the ice shelf collapses in the mid-Holocene.

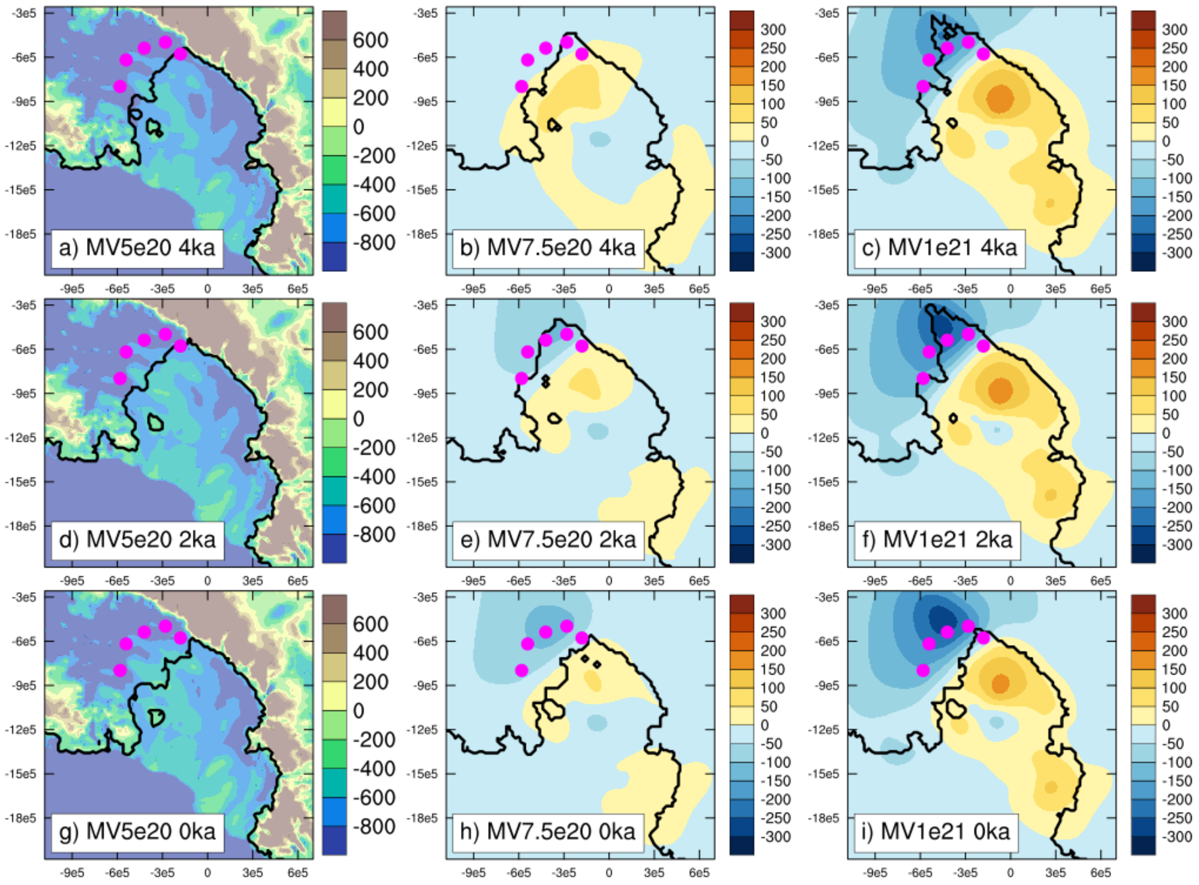


**Fig S7. Late Holocene bed topography differences predicted with varying mantle viscosity.** Left column (a, d, g): Bed topography (m above sea level) of the MV5e20 OT+0.5 simulation at 4, 2 and 0 ka BP, respectively. Middle column (b, e, h): Relative bed topography (m) of the MV7.5e20 simulation to the MV5e20 OT+0.5 simulation at 4, 2 and 0 ka BP, respectively. Right column (c, f, i): Relative bed topography (m) of the MV1e21 simulation to the MV5e20 OT+0.5 simulation at 4, 2 and 0 ka BP, respectively. The grounding line position is indicated by the black line. Magenta circles indicate the West Antarctic subglacial sediment sites.

Global glacioisostatic adjustment (GIA) modelling

Using ice thickness outputs updated every 100 years from the ice sheet model (ISM), we apply a 1D glacioisostatic adjustment (GIA) model, which incorporates radially varying Earth structure. The viscoelastic deformation is solved on a self-gravitating Maxwell viscoelastic Earth with radially varying rheological Structure represented by lithosphere thickness, upper and lower mantle viscosities, and the elastic and density structure is adopted from the seismic Preliminary Reference Earth Model (PREM; *23*). The GIA model is described in detail in Han et al., (2022) (*24*). An example sequence of ice thickness changes for the MV7.5e20 OT+0.5 simulation is shown in Fig S8.

As explained in the main text, we consider two end-member cases of Earth structure relevant for the Ross Sea region: a weak Earth structure model (GIAWE) and a strong Earth structure model, which cover the full spectrum of mantle viscosities estimated for the Ross Sea region (*25*). GIAWE has lithosphere thickness is 60 km, the upper mantle viscosity ranges from 1e18 to 1e19 Pa s and the lower mantle viscosity is ~1e22 Pa s. GIASE has a lithosphere thickness is 90km, the upper mantle viscosity is 5e20 Pa s and the lower mantle viscosity of 5e21 Pa s. We run GIAWE and GIASE experiments for the following experiments: MV5e20, LFR1e24, OT+0.0, +0.3, and +0.5; MV7.5e20, LFR1e24 OT+0.0, +0.3, and +0.5; MV1e21, LFR1e24, OT+0.0, +0.3, +0.5; MV1e21, LFR1e25, OT+0.0, +0.3, and +0.5. This is a total of 24 experiments.

The resulting bed topography changes for the full continent of both models for the ice thickness changes shown in Fig S8 are shown in Fig S9 for the GIAWE and Fig S10 for the GIASE. Because of their overall similarity in terms of solid earth parameters, differences between the ISM and GIASE are substantially lower than those between the ISM and GIAWE.

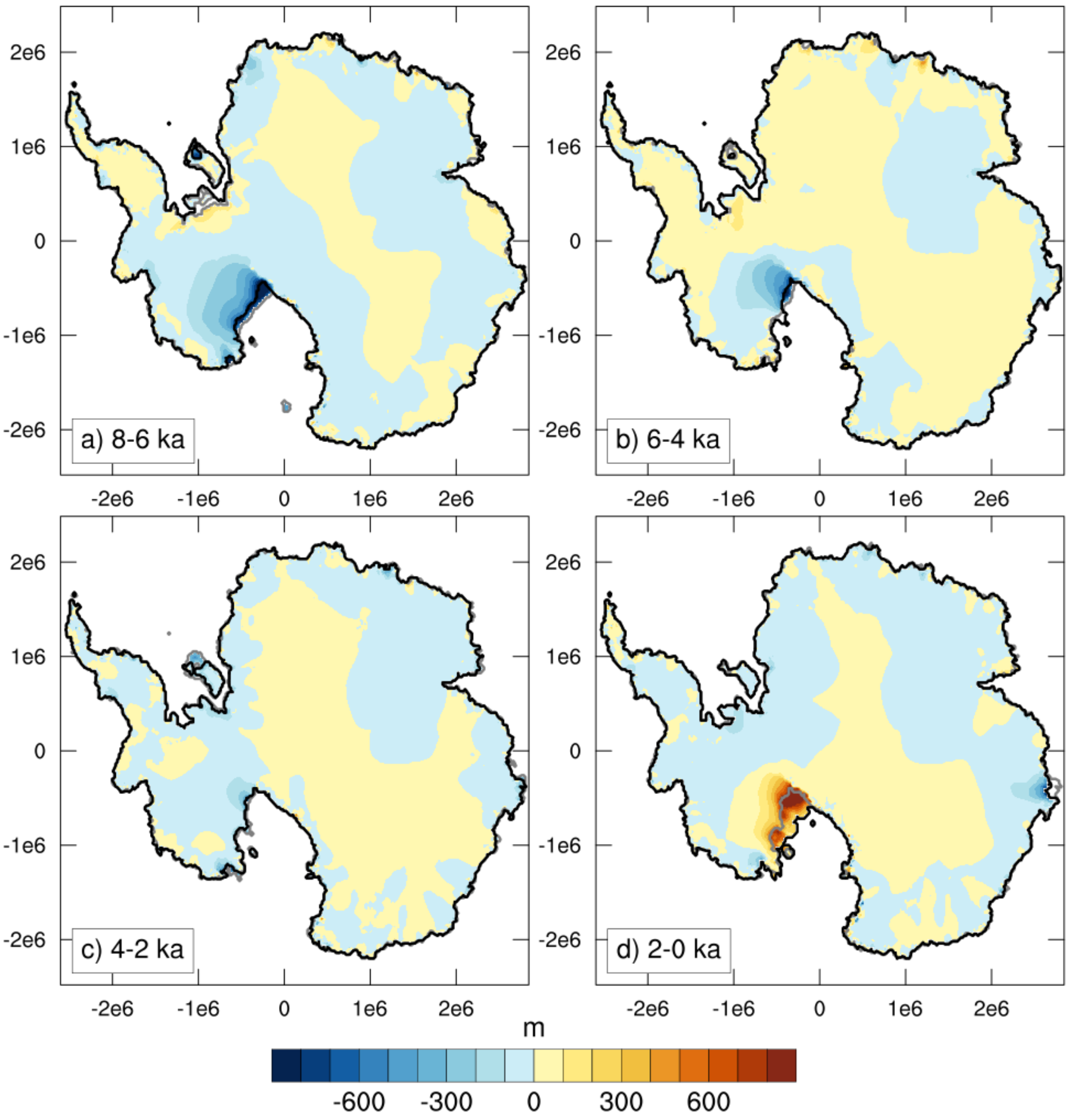


**Fig S8. Example of ice sheet history used as input to the glacioisostatic adjustment (GIA) model.** Ice thickness changes (m) of the MV7.5e20 OT+0.5 simulation from (a) 8 to 6 ka BP, (b) 6 to 4 ka BP, (c) 4 to 2 ka BP, and (d) 2 to 0 ka BP. The gray line indicates the grounding line position at the beginning of each time period, whereas the black line indicates the position at the end.

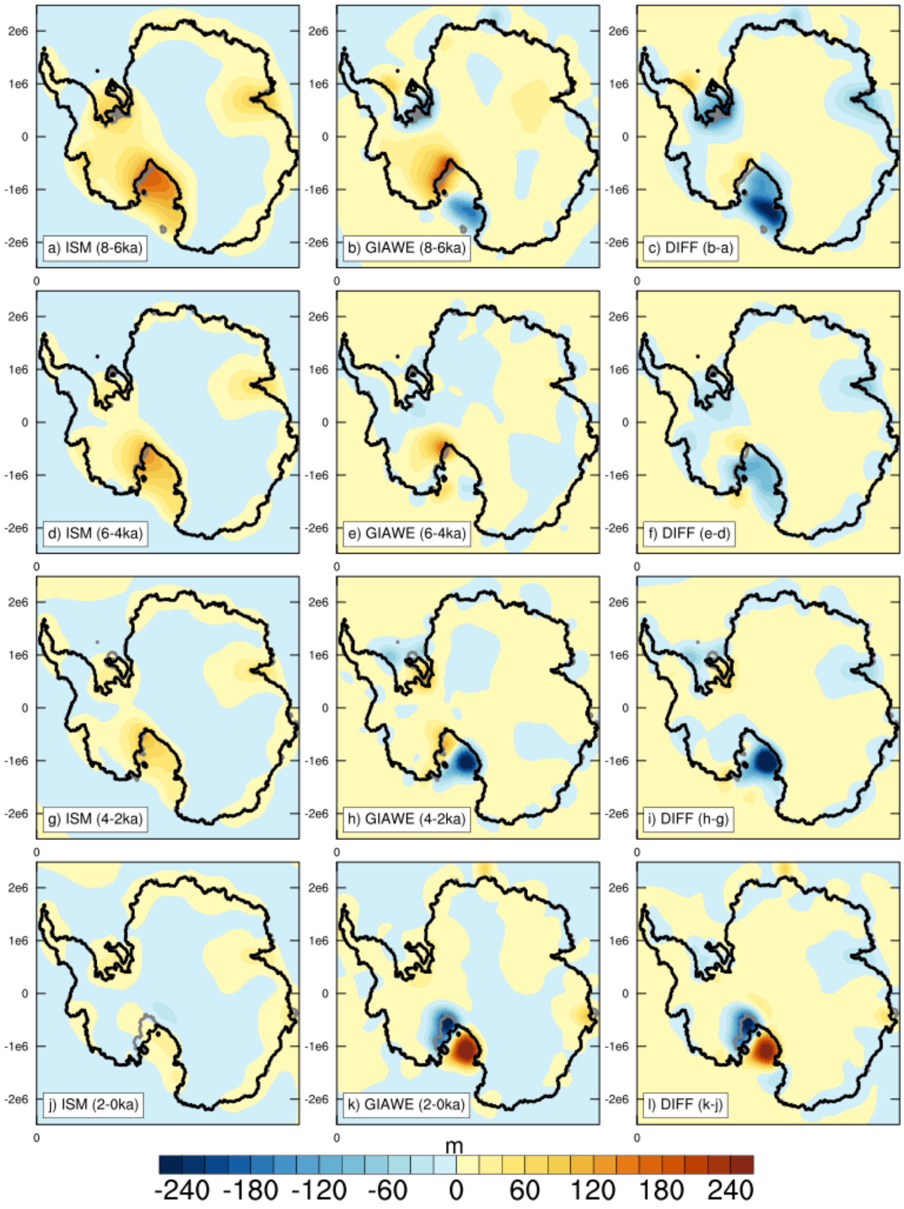


**Fig S9. Holocene bed topography changes predicted by the ice sheet and GIA model with weak Earth structure.** Left column: Bed topography changes (m) of the MV7.5e20 OT+0.5 ice sheet model simulation (ISM) from (a) 8 to 6 ka BP, (d) 6 to 4 ka BP, (g) 4 to 2 ka BP, and (j) 2 to 0 ka BP. Middle column: Bed topography changes (m) of the weak earth GIA model (GIAWE) using ice thickness from the MV7.5e20 OT+0.5 simulation from (b) 8 to 6 ka BP, (e) 6 to 4 ka BP, (h) 4 to 2 ka BP, and (k) 2 to 0 ka BP. Right column: Difference between the bed topography changes of the ISM and the GIAWE from (c) 8 to 6 ka BP, (f) 6 to 4 ka BP, (i) 4 to 2 ka BP, and (l) 2 to 0 ka BP. The gray line indicates the grounding line position at the beginning of each time period, whereas the black line indicates the position at the end.

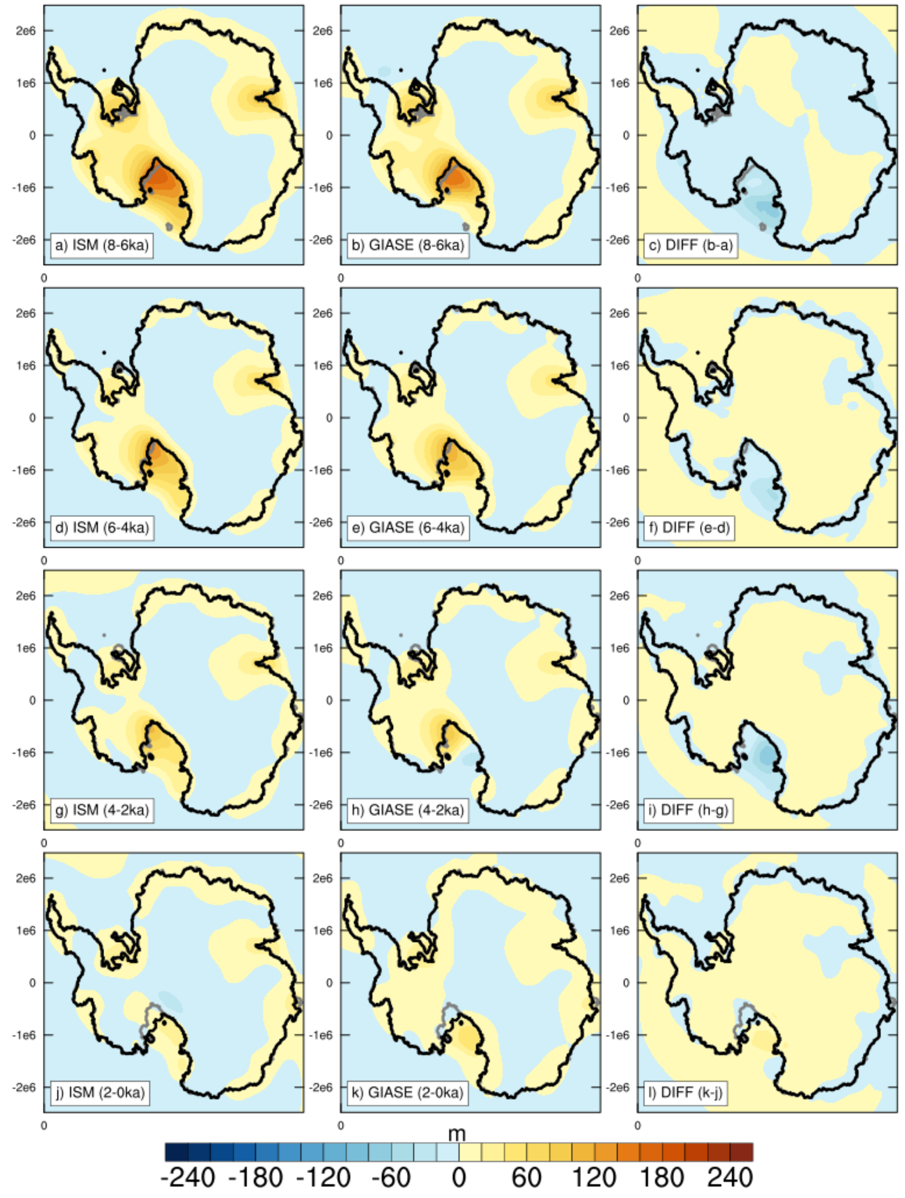


**Fig S10. Holocene bed topography changes predicted by the ice sheet and GIA model with strong Earth structure.** Left column: Bed topography changes (m) of the MV7.5e20 OT+0.5 ice sheet model simulation (ISM) from (a) 8 to 6 ka BP, (d) 6 to 4 ka BP, (g) 4 to 2 ka BP, and (j) 2 to 0 ka BP. Middle column: Bed topography changes (m) of the strong earth GIA model (GIASE) using ice thickness from the MV7.5e20 OT+0.5 simulation from (b) 8 to 6 ka BP, (e) 6 to 4 ka BP, (h) 4 to 2 ka BP, and (k) 2 to 0 ka BP. Right column: Difference between the bed topography changes of the ISM and the GIASE from (c) 8 to 6 ka BP, (f) 6 to 4 ka BP, (i) 4 to 2 ka BP, and (l) 2 to 0 ka BP. The gray line indicates the grounding line position at the beginning of each time period, whereas the black line indicates the position at the end.

Supplementary References


1. Imbrie, J. D., & McIntyre, A. (2006). SPECMAP time scale developed by Imbrie et al. 1984 based on normalized planktonic records (normalized O-18 vs time, specmap. 017). Earth Syst. Sci. Data.

2. Clark, P. U., & Huybers, P. (2009). Interglacial and future sea level. Nature, 462(7275), 856-857.

3. Stanford, J. D., Hemingway, R., Rohling, E. J., Challenor, P. G., Medina-Elizalde, M., & Lester, A. J. (2011). Sea-level probability for the last deglaciation: A statistical analysis of far-field records. Global and Planetary Change, 79(3-4), 193-203.

4. Deschamps, P., Durand, N., Bard, E., Hamelin, B., Camoin, G., Thomas, A. L., ... & Yokoyama, Y. (2012). Ice-sheet collapse and sea-level rise at the Bølling warming 14,600 years ago. Nature, 483(7391), 559-564.

5. Fudge, T. J., Markle, B. R., Cuffey, K. M., Buizert, C., Taylor, K. C., Steig, E. J., ... & Koutnik, M. (2016). Variable relationship between accumulation and temperature in West Antarctica for the past 31,000 years. Geophysical Research Letters, 43(8), 3795-3803.

6. Van Wessem, J. M., Reijmer, C. H., Morlighem, M., Mouginot, J., Rignot, E., Medley, B., ... & Van Meijgaard, E. (2014). Improved representation of East Antarctic surface mass balance in a regional atmospheric climate model. Journal of Glaciology, 60(222), 761-770.

7. Liu, Z., Otto-Bliesner, B. L., He, F., Brady, E. C., Tomas, R., Clark, P. U., ... & Cheng, J. (2009). Transient simulation of last deglaciation with a new mechanism for Bølling-Allerød warming. Science, 325(5938), 310-314.

8. He, F., Shakun, J. D., Clark, P. U., Carlson, A. E., Liu, Z., Otto-Bliesner, B. L., & Kutzbach, J. E. (2013). Northern Hemisphere forcing of Southern Hemisphere climate during the last deglaciation. Nature, 494(7435), 81-85.

9. Golledge, N. R., Clark, P. U., He, F., Dutton, A., Turney, C. S. M., Fogwill, C. J., ... & Carlson, A. E. (2021). Retreat of the Antarctic Ice Sheet during the Last Interglaciation and implications for future change. Geophysical Research Letters, 48(17), e2021GL094513.

10. Ivins, E. R., James, T. S., Wahr, J., O. Schrama, E. J., Landerer, F. W., & Simon, K. M. (2013). Antarctic contribution to sea level rise observed by GRACE with improved GIA correction. *Journal of Geophysical Research: Solid Earth*, *118*(6), 3126-3141.

11. Argus, D. F., Peltier, W. R., Drummond, R., & Moore, A. W. (2014). The Antarctica component of postglacial rebound model ICE-6G_C (VM5a) based on GPS positioning, exposure age dating of ice thicknesses, and relative sea level histories. *Geophysical Journal International*, *198*(1), 537-563.

12. Simms, A. R., Lisiecki, L., Gebbie, G., Whitehouse, P. L., & Clark, J. F. (2019). Balancing the last glacial maximum (LGM) sea-level budget. *Quaternary Science Reviews*, *205*, 143-153.

13. Todd, C., Stone, J., Conway, H., Hall, B., & Bromley, G. (2010). Late Quaternary evolution of Reedy Glacier, Antarctica. Quaternary Science Reviews, 29(11-12), 1328-1341.

14. Spector, P., Stone, J., Cowdery, S. G., Hall, B., Conway, H., & Bromley, G. (2017). Rapid early-Holocene deglaciation in the Ross Sea, Antarctica. Geophysical Research Letters, 44(15), 7817-7825.

15. Hillebrand, T. R., Stone, J. O., Koutnik, M., King, C., Conway, H., Hall, B., ... & Gillespie, M. K. (2021). Holocene thinning of Darwin and Hatherton glaciers, Antarctica, and implications for grounding-line retreat in the Ross Sea. The Cryosphere, 15(7), 3329-3354.

16. Stutz, J., Eaves, S., Norton, K., Wilcken, K. M., Moore, C., McKay, R., ... & Johnson, K. (2023). Inland thinning of Byrd Glacier, Antarctica, during Ross Ice Shelf formation. Earth Surface Processes and Landforms.

17. Morlighem, M., Rignot, E., Binder, T., Blankenship, D., Drews, R., Eagles, G., ... & Young, D. A. (2020). Deep glacial troughs and stabilizing ridges unveiled beneath the margins of the Antarctic ice sheet. Nature geoscience, 13(2), 132-137.

18. Bentsen, M., Bethke, I., Debernard, J. B., Iversen, T., Kirkevåg, A., Seland, Ø., ... & Kristjansson, J. E. (2013). The Norwegian Earth System Model, NorESM1-M–Part 1: description and basic evaluation of the physical climate. Geoscientific Model Development, 6(3), 687-720.

19. Adusumilli, S., Fricker, H. A., Medley, B., Padman, L., & Siegfried, M. R. (2020). Interannual variations in meltwater input to the Southern Ocean from Antarctic ice shelves. Nature geoscience, 13(9), 616-620.

20. Bueler, E. D., Lingle, C. S., & Brown, J. (2007). Fast computation of a viscoelastic deformable Earth model for ice-sheet simulations. Annals of Glaciology, 46, 97-105.

21. Neuhaus, S. U., Tulaczyk, S. M., Stansell, N. D., Coenen, J. J., Scherer, R. P., Mikucki, J. A., & Powell, R. D. (2021). Did Holocene climate changes drive West Antarctic grounding line retreat and readvance?. *The Cryosphere*, *15*(10), 4655-4673.

22. Mouginot, J., B. Scheuchl, and E. Rignot. (2017). MEaSUREs Annual Antarctic Ice Velocity Maps, Version 1 [Data Set]. Boulder, Colorado USA. NASA National Snow and Ice Data Center Distributed Active Archive Center. https://doi.org/10.5067/9T4EPQXTJYW9.

23. Dziewonski, A. M., & Anderson, D. L. (1981). Preliminary reference Earth model. Physics of the earth and planetary interiors, 25(4), 297-356.

24. Han, H. K., Gomez, N., & Wan, J. X. W. (2022). Capturing the interactions between ice sheets, sea level and the solid Earth on a range of timescales: a new “time window” algorithm. Geoscientific Model Development, 15(3), 1355-1373.

25. Whitehouse, P. L., Gomez, N., King, M. A., & Wiens, D. A. (2019). Solid Earth change and the evolution of the Antarctic Ice Sheet. Nature communications, 10(1), 503.
